# Principles and functions of condensate modifying drugs

**DOI:** 10.3389/fmolb.2022.1007744

**Published:** 2022-11-22

**Authors:** Avinash Patel, Diana Mitrea, Vigneshwaran Namasivayam, Mark A. Murcko, Michael Wagner, Isaac A. Klein

**Affiliations:** ^1^ Dewpoint Therapeutics GmbH, Dresden, Germany; ^2^ Dewpoint Therapeutics, Boston, MA, United States; ^3^ Dewpoint Therapeutics GmbH, Frankfurt, Germany

**Keywords:** condensatopathy, dissolvers, inducers, localizers, morphers, biomolecular condensates, condensate modifying drugs (c-mods)

## Abstract

Biomolecular condensates are compartmentalized communities of biomolecules, which unlike traditional organelles, are not enclosed by membranes. Condensates play roles in diverse cellular processes, are dysfunctional in many disease states, and are often enriched in classically “undruggable” targets. In this review, we provide an overview for how drugs can modulate condensate structure and function by phenotypically classifying them as dissolvers (dissolve condensates), inducers (induce condensates), localizers (alter localization of the specific condensate community members) or morphers (alter the physiochemical properties). We discuss the growing list of bioactive molecules that function as condensate modifiers (c-mods), including small molecules, oligonucleotides, and peptides. We propose that understanding mechanisms of condensate perturbation of known c-mods will accelerate the discovery of a new class of therapies for difficult-to-treat diseases.

## 1 Introduction

Biomolecular condensates are membrane-less cellular compartments with distinct physiochemical properties. Communities of biomolecules including nucleic acids, metabolites, and proteins assemble *via* extensive interaction networks, on length scales of nanometers to micrometres, locally concentrating selected components. The physicochemical properties of a condensate arise from the emergent properties of the molecular community (reviewed in (Banani et al., 2017; Lyon et al., 2021a; Mitrea et al., 2022)). Importantly, proteins containing long intrinsically disordered regions (IDRs) are highly enriched in condensates. Due to the lack of well-defined secondary and tertiary structures, IDRs are not amenable to traditional structure-guided drug discovery, and therefore have been historically considered undruggable (Biesaga et al., 2021).

Condensates concentrate factors involved in shared processes, leading to the regulation of a wide array of cellular functions such as signaling, stress adaptation, and gene regulation. The physicochemical properties and functions of condensates are dictated by their composition and the structural and conformational features of the extended molecular network between components. Therefore, changes in environmental conditions, genetic modifications, post translational modifications (PTMs) and other processes that alter this balance regulate condensates (Lyon et al., 2021b). Aberrant disruption of any one of these parameters can lead to a pathogenic cellular state, so the same pathogenic endpoint can be reached by deregulating a condensate *via* disparate mechanisms. In these cases, condensates serve as integrating nodes of disease (Mitrea et al., 2022). We define a “condensatopathy” as an aberration of a condensate that drives a specific disease phenotype. Notably, as defined, a condensatopathy can be causative or strongly associated with a particular disease (Mitrea et al., 2022). Thus, condensates have emerged as appealing drug targets. They hold the promise of effectively targeting undruggable proteins, and/or deploying a single therapeutic strategy against a wide array of genotypes with the same disease phenotype.

On the road to leveraging condensate biology to develop a new class of condensate modifying drugs (c-mods), several challenges and questions remain. *What chemical modalities can be c-mods? Do c-mods need to interact directly with the target(s) in condensates? Will targeting pathways that regulate the condensation process be a suitable strategy? Should single or multiple components within a condensate community be targeted? What are the phenotypic readouts of condensates that can be used for drug screening?* In this review we will draw on examples in the literature (Table 1) to address some of these questions. We will describe some of the observable condensate changes for known drugs (approved or currently in development). We will combine the known mechanisms of action of these c-mods with our current knowledge of condensate biology and biophysics to propose chemical design principles that might be relevant for the discovery of breakthrough medicines.

**TABLE 1:** c-mod therapeutic modalities. A table collating the different classes of c-mods (dissolvers, inducers, localizers, and morphers), the condensate target, the representative chemical modality (small molecule, peptide, anti-sense oligo), the molecular target (if reported in the study) and the reference of the study that reported the observation that is reminiscent of a condensate modification.

cmod class	target condensate	c-mod	Modality	Target	Reference
**Dissolvers**	Stress granules	Planar compounds (Mitoxantrone, daunorubicin, quinacrine, pyrvinium)	small molecule	Unknown RNA-protein interaction	Fang et al. (2019)
		ISRIB and related compounds	small molecule	eIF2b	Sidrauski et al. (2015), Zyryanova et al. (2018, 2021)
		Nobiletin	small molecule	unknown	Jahan et al. (2022)
		GSK2334470	small molecule	PI3K	Heberle et al. (2019)
		Wortmannin	small molecule	PI3K	
		AZD8055	small molecule	mTOR	
		Everolimus	small molecule	mTORC1	
		GSK-626616	small molecule	Dyrk3	Rai et al. (2018)
		RK33	small molecule	DDX3	Cui et al. (2020)
		PJ34	small molecule	PARP	Catara et al. (2017)
		GAP161	peptide	G3BP1	Zhang et al. (2012)
		ION363	antisense oligo	FUS	Korobeynikov et al. (2022)
	chemoresistance	SI-2	small molecule	SRC3	Liu et al. (2021)
		Tamoxifen	small molecule	MED1	Klein et al. (2020)
	Tau	Myricetin	small molecule	Tau-ATG5 interaction	Dai et al, (2021)
	p53 mutant	ADH-1, ADH-6	peptide	p53	Palanikumar et al. (2021)
	Viral factories	NIP-V	peptide	SARS2-NP	Wang et al. (2021)
	CCG-repeat expansion bodies	ASO-CCG	antisense oligo	FMR1	Derbis et al. (2021)
	C9ORF72 expansion bodies	c9ASO	antisense oligo	C9ORF72	Cook et al. (2020)
					
**Inducers**	Proteolysis inducers	BI-3802	small molecule	BCL6	Słabicki et al. (2020)
		Rho-rock inhibitors (Y27632, Y39983)	small molecule	INAVA	Chang et al. (2021)
		G007-LK, XAV939	small molecule	Tankyrase	Martino-Echarri et al. (2016)
		Sulphoraphane	small molecule	unknown	Bernkopf et al. (2018)
		Lorecivivint	small molecule	CLK2, DYRK1A	Deshmukh et al. (2019)
		PCG	small molecule	Brd4	Wang et al. (2022)
		Guanabenz	small molecule	Gαi2	Miete et al. (2022)
		Aggregon	peptide	Conductin/ Axin2	Bernkopf et al. (2019)
		Gαi2 peptide	peptide	Conductin/ Axin2	Miete et al. (2022)
	DNA repair bodies	Camptothecin, Mitoxantrone, Etoposide	small molecule	DNA topoisomerase I and II	Zhao et al. (2012)
		Olaparib	small molecule	PARP	Michelena et al. (2018)
	Cajal Body	Nusinersen ASO	antisense oligo	SMA	Berciano et al. (2020)
	Synaptic densities	NA-1, AVLX-144	peptide	PSD95	Christensen et al. (2021)
					
**Localizers**	Nucleolus	Selinexor (KPT-330)	small molecule	XPO1/ CRM1	Gu et al. (2016)
		Avrainvillamide	small molecule	NPM1	Andresen et al. (2016)
		Platinum compounds (Oxaliplatin, cisplatin and carboplatin)	small molecule	unknown	Sutton et al. (2019)
		BMH21	small molecule	Pol1	Colis et al. (2014)
		UNC6934	small molecule	NSD2	Dilworth (2021)
	EML4-ALK chemoresistance	Crizotinib	small molecule	EML4-ALK	Gonzalez-Martinez (2022)
	DNA repair	Tropotecan	small molecule	DNA topoisomerase	Mo (2002)
	Aberrant EWS-WT1, EWS-FLI1 transcriptional condensate & nucleolus	Lurbinectedin, Trabectedin	small molecule	EWSR1 fusions	Gedminas (2022), Harlow (2016)
					
**Morphers**	Viroplasm	Cyclopamine	small molecule	M2-1	Risso-Ballester (2021)

## 2 Classification of c-mods

**FIGURE 1:**
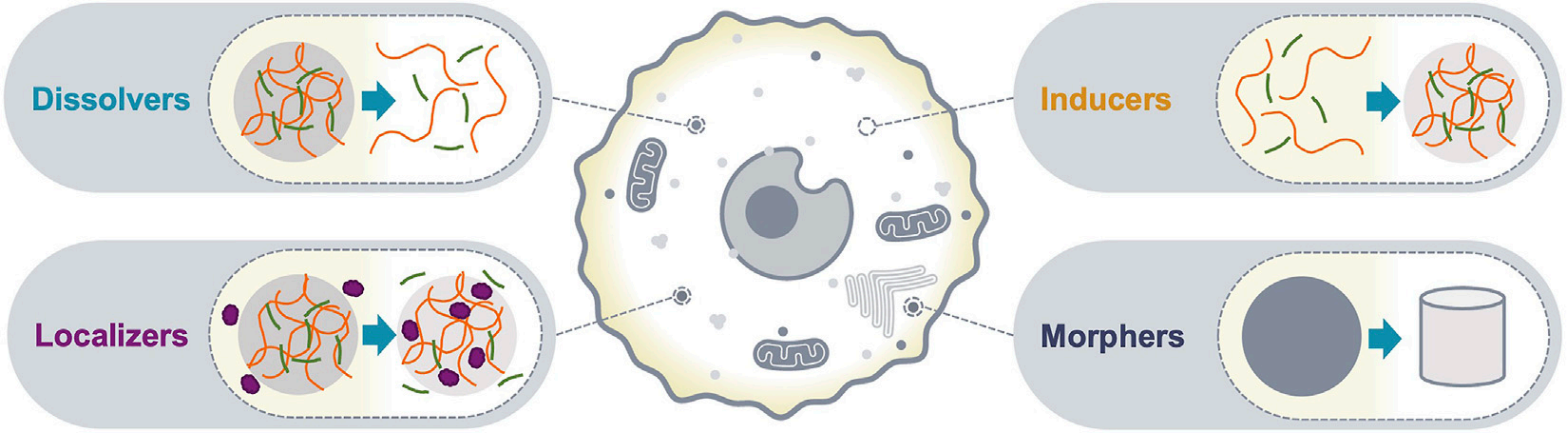
c-mod classes. A schematic representation of the optical phenotypes of condensate modification used to classify c-mods. Dissolvers drive dissolution of an existing condensate. Inducers cause assembly of a new condensate. Localizers alter the localization of specific condensate community members. Morphers alter the physiochemical properties of an existing condensate.

Biomolecular condensates with sizes above the diffraction limit can be detected as bright puncta using conventional fluorescence microscopy; the number, shape and relative fluorescence intensities can be quantified and interpreted as the optical phenotype of the condensate. Changes in the optical phenotype of a condensate are a consequence of altered composition and/or organization of its molecular community, and typically correlate with changes in functional output. Therefore, c-mods can be classified based on the phenotypic change of condensates in imaging assays. Here, we classify c-mods in four categories (Figure 1): 1) dissolvers, dissolve condensates, 2) inducers, induce the formation of condensates, and 3) localizers, alter the sub-cellular localization of condensate community members, and 4) morphers, change the morphology of existing condensates by altering their biophysical properties (e.g., material properties). We will discuss each of these categories below and provide prototypes from the literature. These examples reveal potential mechanisms of action and modalities that may be deployed for future c-mod development.

*What chemical modalities can be c-mods?*

**FIGURE 2:**
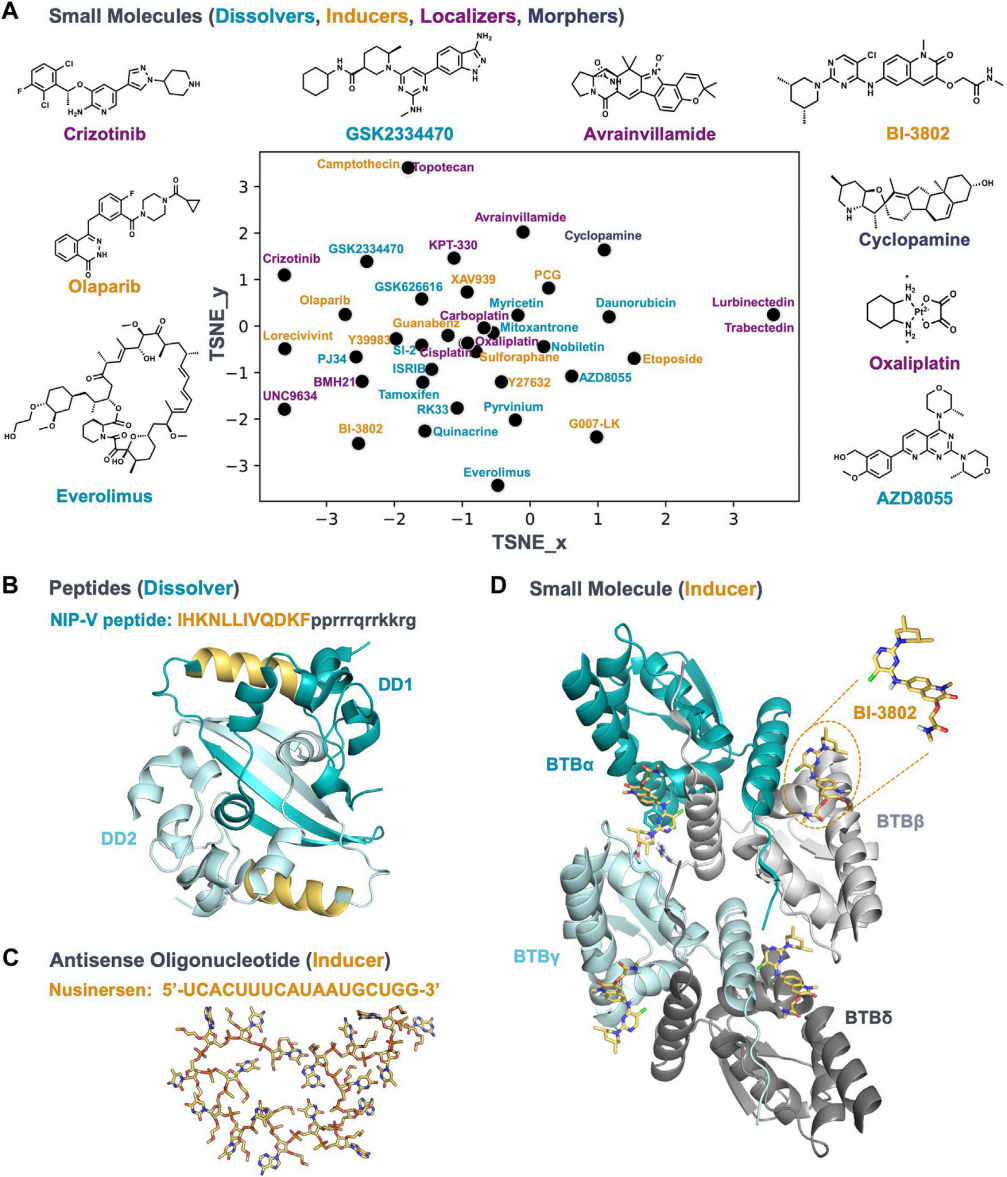
A chemically diverse set of bioactive molecules act as c-mods. **(A)** The default t-SNE projection of small molecules by applying rdkit fingerprints; the molecules are reported in Table 1; the molecule names are colored according to the c-mod classes shown in Figure 1. The 2D structure of representative small molecules are shown. **(B)** A representative of the dissolver c-mod class, The NIP-V peptide sequence (gold) binds to the dimerization domain (DD) of the SARS2-NP (PDB ID: 7C22), A Tat-fusion (in black) was included in NIP-V to facilitate cellular uptake. **(C)** and **(D)** Representatives of the inducer c-mod class. **(C)** Nusinersen ASO structure, downloaded from PubChem, was energy minimized and the lowest energy conformer was searched using Molecular Operating Environment (MOE 2020.0901). **(D)** The cryo-EM structure of BCL6-BTB filament in complex with BI-3802 (PDB ID: 6XMX) and binding conformation of BI-3802 in BCL6.

A variety of mechanisms can alter the condensate community; therefore c-mods are not limited to small molecules. New chemical modalities, such as chimeric protein degraders, peptides, and oligonucleotides can function as c-mods as well. In the following paragraphs we will give a few examples where different chemical modalities have been described in the context of condensates. The small molecules we cover are plotted in Figure 2A to show the range of chemical diversity. Examples of other modalities are shown in Figures 2B,C.

BOX 1 **Considerations for c-mods screening**

Condensates are dynamic, integrating nodes of many cellular mechanisms and are thus affected by numerous factors. This has several advantages but also many disadvantages for drug discovery. Many compounds targeting essential and generic cellular processes (e.g., cytoskeleton, proteolytic degradation machineries, metabolism, nuclear transport, intracellular ionicity, osmolarity homeostasis, translation, and transcription) will modulate condensates, and will therefore be false c-mod positives in high-throughput screens only focusing on condensate observable phenotype. Secondary counter screens and downstream functional screens need to be carefully designed in disease relevant cell types to detect and filter out false c-mods. Some c-mod mechanisms of action will be cell type specific. Not selecting the correct cell type could also lead to false positives and misinterpretations. Careful and diligent downstream assays will help in selecting and progressing c-mods with a higher likelihood for success in the drug discovery value chain.

The c-mods that do not progress toward the clinic should not be neglected. They are highly valuable molecular tools useful for dissecting the pathological mechanism of condensates. In traditional drug discovery often the hits that fail to progress through the different drug discovery processes are filtered out and neglected. The mechanisms underlying the disease causality of most of the condensates are still in relatively early stages. Therefore, a molecular toolbox of c-mods that have a phenotypic effect on condensates will enable the exploration of the functional link to diseases. For example, the planar compounds that lack specificity for condensates owing to their general nucleic acid intercalation mechanism of action will unlikely be drug candidates. However, they will make good tool compounds for SGs in ALS models. They may serve as positive controls for future c-mod screens and may be used to establish the role of SGs in disease relevant functional assays using patient derived neuronal cell models.

### 2.1 Dissolvers

Studies have shown that some condensatopathies such as the formation or persistence of certain condensates are not required for cellular physiology but instead drive disease pathologies (Patel et al., 2015; Alberti and Hyman, 2021). Dissolver c-mods either dissolve or prevent the formation of pathological condensates.

#### 2.1.1 Persistent condensates in ALS

Compounds with planar moieties such as mitoxantrone, daunorubicin, quinacrine, and pyrvinium (Table 1 and plotted in Figure 2A) are effective at dissolving persistent stress granules (SGs) in cells. SGs concentrate many proteins that have mutations genetically linked to driving pathobiological mechanisms of amyotrophic lateral sclerosis (ALS), a fatal neurodegenerative disease mostly affecting the motor neurons. (Dudman and Qi, 2020). SGs are also composed of many disease-associated intrinsically disordered RNA-binding proteins such as TDP43 and FUS (Nedelsky and Taylor, 2022), and their persistence is implicated in the pathophysiology of ALS (Fang et al., 2019; Dudman and Qi, 2020). Unresolved persistent stress granules evolve into disease-associated pathological aggregates, such as those seen in spinal cord motor neurons of ALS patients, resulting in a gain and/or loss-of-function of the sequestered molecular community (Alberti and Hyman, 2021; Nedelsky and Taylor, 2022). Two scenarios for targeting these SG condensatopathies might have clinical relevance: 1) specifically dissolving the persistent SGs; 2) inhibiting the formation of SGs. Dissolution of the persistent condensates is more attractive because it is more likely to hinder the disease progression in patients already presenting the disease symptoms. Planar compounds mitoxantrone and pyrvinium dissolve persistent TDP43 condensates, whereas non-planar compounds are mostly able to inhibit condensate formation. Planar compounds can bind to nucleic acids and potentially to IDRs, leading to dissolution of SGs (Fang et al., 2019). Mitoxantrone has also been demonstrated to be effective under a range of cellular stresses in disease-relevant motor neurons differentiated from patient-derived induced pluripotent stem cells (iPSCs). Furthermore, mitoxantrone reduces neuronal death of mouse primary neurons bearing disease-associated TDP43 mutant condensates. Therefore, planar compounds like mitoxantrone may function as dissolver c-mods for persistent SG condensatopathies in ALS/FTD.

The small molecule ISRIB (Table 1 and plotted in Figure 2A) is a dissolver c-mod for SGs (Sidrauski et al., 2015) and has been proven neuroprotective in several disease models, such as cellular models of ALS (Bugallo et al., 2020) and mouse models of traumatic head injury (Chou et al., 2017) and Down syndrome (Zhu et al., 2019). Two close analogues of ISRIB are currently in Phase 1 clinical trials for the treatment of ALS. Upon cellular stress, SGs assemble to concentrate translationally stalled proteins and RNA. Phosphorylation of the translational initiation factor subunit eIF2α reduces overall protein translation. ISRIB reverses the phosphorylated eIF2α-dependent SG formation and restores protein translation (Sidrauski et al., 2015), resulting in both dissolution of persistent SGs and prevention of their formation. Recent mechanistic studies show that ISRIB allosterically modulates the interaction between phosphorylated eIF2α and eIF2B and restores the guanine nucleotide exchange activity of eIF2B (Zyryanova et al., 2021). EIF2α/B are known to be part of the SG condensate community (Anderson and Kedersha, 2006). Therefore ISRIB (and analogues) modulates the interactions within the condensate community to drive SG dissolution and reverse the aberrant effects of phosphorylated eIF2α (Zyryanova et al., 2021).

Another small molecule Nobiletin (Table 1 and plotted in Figure 2A) was shown to dissolve SGs and rescue neuronal health in human iPSC derived neuronal models of ALS. The exact mode of action for dissolution is not reported (Jahan et al., 2022), but based on the optical phenotype, we argue it is a dissolver c-mod.

#### 2.1.2 Condensate-mediated drug resistance in cancer

Dissolver c-mods may also be useful for treating condensate-mediated drug resistance to existing cancer therapies. Tamoxifen is an estrogen receptor (ER) agonist used to treat breast cancer; mutations in ER and overexpression of the transcription co-activator MED1 lead to acquired drug resistance and poor prognosis (Nagalingam et al., 2012). ER condenses in a ligand-dependent manner with MED1, causing aberrant gene activation and thereby driving disease (Boija et al., 2018). Treatment of *in vitro* reconstituted condensates of wild type ERαand MED1-IDR with tamoxifen lead to ejection of ERα. However, ejection of ERα from condensates was inhibited when ERα contained a drug resistance mutation or under conditions of 4-fold higher MED1-IDR concentrations, thereby stimulating the drug resistant state. Similarly, tamoxifen reduced the size of MED1 nuclear condensates in breast cancer cell lines expressing low levels, but not in resistant cells expressing high levels of MED1 (Klein et al., 2020). The drug resistance due to MED1 overexpression correlated with poorer partitioning of FLTX1—a tamoxifen analogue—into *in vitro* condensates (Klein et al., 2020). Together, these results support a model where knowledge of the correlation between the drug efficacy and target condensate biology can inform new strategies for drug development. For example, in the case of tamoxifen, the drug hunter could optimize drug partitioning into the MED1-rich condensates or use a combination therapy by including a dissolver that destabilizes these drug-resistant condensates.

Combination therapy with a traditional drug and a dissolver c-mod may also have therapeutic benefit in drug-resistant multiple myeloma. Overexpression of the histone methyltransferase NSD-2 and steroid receptor coactivator-3 (SRC-3) has been implicated as a driver of acquired drug resistance in multiple myeloma. Recent findings showed that NSD-2 and SRC-3 are part of the same condensate community that drives chemoresistance to the standard of care, bortezomib. (Liu et al., 2021). Bortezomib is a proteasomal inhibitor and a likely mechanism of action is through preventing the degradation of pro-apoptotic factors driving the immortality of the tumour cells (Field-Smith et al., 2006). The IDR domains of NSD-2 and SRC-3 interact to form aberrant condensates (Liu et al., 2021). A small molecule SI-2 (Table 1 and plotted in Figure 2A) dissipates these bortezomib-resistant condensates by disrupting this interaction, thereby restoring bortezomib sensitivity in model systems (Liu et al., 2021). Condensate dissolution translates to improved sensitivity to borteozomib in cellular and animal models of multiple myeloma. Therefore, condensate dissolver c-mods that block NSD-2 and SRC-3 from interacting in condensates may present a new therapeutic opportunity for drug resistance in multiple myeloma.

#### 2.1.3 Condensate-mediated viral pathogenicity

A dissolver peptide c-mod that interacts with the severe acute respiratory syndrome coronavirus 2 (SARS-CoV-2) nucleocapsid protein has been proposed as a promising therapeutic treatment for rescuing innate antiviral immunity during SARS-CoV-2 infection (Wang et al., 2021). The SARS-CoV-2 nucleocapsid protein (SARS2-NP) condenses with the viral genomic RNA to promote virion packaging and replication (Lu et al., 2021). The dimerization domain of the SARS2-NP is indispensable for condensation and association with viral RNA, and subsequently for suppression of the innate antiviral immune response *in vitro* and *in vivo*. For instance, acetylation of Lys375 of SARS2-NP, which is adjacent to the dimerization domain, leads to inhibition of condensation, and reduced suppression of the innate immune response. Several peptides were designed to block the dimerization of SARS2-NP (Wang et al., 2021). Figure 2B shows the structure of this dimerization interface: in the dimerization domain, two β strands are associated with and stabilized by several helices. A specific peptide (NIP-V) from the helix sitting adjacent to the β strands (Table 1 and shown in gold in Figure 2B) dissolved condensates *in vitro*, inhibited condensation in living cells, and enhanced the innate antiviral response in mice infected with VSV-NP (Wang et al., 2021). Condensates are targets for several viral pathogens (Etibor et al., 2021); therefore, peptides as c-mod dissolvers could be attractive anti-viral therapeutic strategies (Wang et al., 2021).

### 2.2 Inducers

Condensates can inhibit biochemical reactions by sequestering and inactivating cellular factors. Thus, inducer c-mods may trigger the formation of a condensate to sequester disease-driving factors. Alternatively, condensates have been shown to increase biochemical reaction rates, and such c-mods might also be used to accelerate or initiate biochemical reactions (Peeples and Rosen, 2021).

#### 2.2.1 Aberrant Cajal bodies in spinal muscular atrophy

Antisense oligonucleotide (ASO) Nusinersen (Table 1 and Figure 2C) an approved therapy for SMA (reviewed in (Edinoff et al., 2021)), acts as an inducer c-mod. SMA is a devastating neuromuscular disease resulting in degradation and excessive loss of alpha motor neurons (αMNs) in the spinal cord. αMN degeneration is caused by the reduced expression of survival motor neuron (SMN) due to deletion or mutation of the SMN1 gene. Functional SMN plays a key role in spliceosome biogenesis (Berciano et al., 2020; Edinoff et al., 2021). A second gene SMN2 produces an alternative spliced isoform expressed as a truncated, non-functional form of the SMN protein (SMNΔ7). Nusinersen is an ASO targeted at the SMN2 transcript splicing to rescue the production of functional SMN protein expression and downstream spliceosomal biogenesis. It was proposed that the underlying mechanism of Nusinersen is through the induction of nuclear condensates (Cajal bodies), resulting in enhanced pre-mRNA transcription, splicing and nuclear export of mature mRNA for translation (Berciano et al., 2020). The results are supported by studies in the αMNs of mice expressing SMNΔ7, showing that Nusinersen normalized SMN expression and motor function through induction of Cajal bodies (Berciano et al., 2020). Although Nusinersen is an ASO targeting the SMN2 gene, the underlying mechanism induces Cajal bodies to rescue disease pathology, indicating it behaves as an inducer c-mod.

#### 2.2.2 Condensate-mediated proteolysis in cancer

Condensates serve as molecular intermediates for proteasomal- and autophagy-mediated degradation, and thus may participate in or catalyze therapeutic degradation (reviewed in (Lei et al., 2021)). We discuss several small molecules for targeted protein degradation, currently at different clinical stages of development, that may act as inducer c-mods.

BI-3802 (Table 1 and shown in Figures 2A,D) is a small molecule that induces condensation of the oncogenic transcriptional repressor B cell lymphoma 6 (BCL6) (Słabicki et al., 2020). BCL6 overexpression drives the pathobiology of many B cell malignancies, including diffuse large B cell lymphoma (DLBCL). BCL6 represses key oncogenic repressor genes such as cell cycle checkpoint genes. BCL6 is an attractive therapeutic target for B cell lymphomas since its inhibition results in activation of target genes that rescue homeostatic balance and reduce proliferation (Hatzi and Melnick, 2014). Direct binding of BI-3802 to the BCL6 dimers (Figure 2D) induces formation of reversible, bright cellular puncta in treated cells expressing eGFP-BCL6 (Słabicki et al., 2020). These condensates recruit E3 ubiquitin ligase, eventually leading to the proteasomal degradation of the BCL6 condensate. Condensation was demonstrated to be important for degradation by a range of cellular and biochemical assays interrogating the structure-activity relationship (SAR) of BI-3802. Particularly, mutations in BCL6 that compromise condensate induction by BI-3802 abolished the degradation and associated reduction in cellular proliferation (Słabicki et al., 2020). Additionally, the inducer c-mod inhibitors, such as BI-3802, are more potent than classical BCL6 inhibitors.

In cellular models of chronic inflammatory bowel disease (IBD), small molecule inhibitors induced condensates of innate immune activator (INAVA) to promote ubiquitin-mediated degradation. INAVA plays a key role in maintaining epithelial homeostasis and regulates inflammatory signaling. Genetic association studies have identified a link between IBD and lower expression of INAVA, which leads to dysfunction in epithelial cell barrier function and aberrant inflammatory signaling, resulting in disease. INAVA usually localizes at cell-cell junctions, but forms cytoplasmic condensates as a response to active inflammatory signaling. Induced cytosolic condensates promote ubiquitination of sequestered effectors to promote inflammatory signaling (Luong et al., 2018). Like inhibitors of BCL6, the inhibitors of the Rho-ROCK pathway promoted the recruitment of an E3 ubiquitin ligase to INAVA cytosolic condensates and mediated ubiquitination and proteasomal degradation (Chang et al., 2021).

Tankyrase inhibitors are a class of small molecule inhibitors that lower the elevated beta-catenin levels implicated in the proliferative pathobiology of colorectal cancer (Mizutani et al., 2018; Zhang and Lum, 2018) and appear to induce condensates. Inhibition of tankyrase, a poly-ADP ribose polymerase (PARP), promotes the formation of a degradation complex. The degradation complex consists of protein regulators that drive degradation through inducing PTMs such as phosphorylation and ubiquitination. The cellular phenotype of an induced destruction complex is reminiscent of condensates when visualized by fluorescent microscopy (Roche et al., 2014; Martino-Echarri et al., 2016). Additionally, induction of a distinct DACT1 condensate is implicated to promote the formation of destruction complex condensates in disease models of breast and prostate cancer bone metastasis (Esposito et al., 2021). Therefore, Tankyrase inhibitors function as inducer c-mods of the beta-catenin destruction complex condensates that regulate Wnt signal transduction in colorectal cancer. Similarly, short peptide inducers of beta-catenin destruction complex condensates have been shown to reduce the levels of beta-catenin in cellular and *in vivo* models of colorectal cancer (Bernkopf et al., 2019; Miete et al., 2022). The short peptide Gαi2, a subunit of the trimeric G-protein, induces the formation of the destruction complex by interfering with the aggregation of conductin—the scaffold protein for the destruction complex (Bernkopf et al., 2019; Miete et al., 2022). A further proof-of-principle for down regulation of beta-catenin by inducer c-mods is Guanabenz (Table 1 and plotted in Figure 2A), an FDA-approved drug for hypertension. Guanabenz is shown to induce beta-catenin destruction condensates through Gαi2 activation, and therefore could be used as a potential therapy in cancer driven by overactive beta-catenin (Miete et al., 2022).

#### 2.2.3 Condensate sequestration of pathological proteins in cancer

A natural product sulforaphane (Table 1 and plotted in Figure 2A) acts as an inducer c-mod for beta-catenin condensates in the nucleus, resulting in inhibition of the Wnt pathway, independent of beta-catenin degradation (Bernkopf et al., 2018). A likely mechanism for inactivation is non-productive sequestration of beta-catenin within condensates.

There are several reports of inducer c-mods; however, their underlying condensate mechanisms of action are unclear. Lorecivivint (Table 1 and plotted in Figure 2A) is a Wnt pathway inhibitor, independent of beta-catenin modulation, currently in phase 3 clinical trials for treating osteoporosis. Lorecivivint, a dual kinase inhibitor of CLK2 and DRK1A, induces splicing factor condensates, which might be the mechanism underlying a transcriptional rescue of an altered Wnt signaling in cellular models of osteoporosis (Deshmukh et al., 2019). The natural product PCG (Table 1 and plotted in Figure 2A) has been shown to induce condensation of the transcription co-activator BRD4, which is implicated in several cancers and infectious diseases (Wang et al., 2022).

Taken together, inducer c-mods are an attractive therapeutic strategy for several reasons. First, they can induce biochemical processes necessary for the rescue of cellular homeostasis in disease conditions. Second, they can inactivate a condensate community by sequestration. Third, they can promote proteolytic degradation.

### 2.3 Localizers

Condensate structure and function are directly linked to the composition of the condensate community. Erroneous localization of proteins away from their physiological condensate community, or within a non-native condensate can drive pathological disease mechanisms. Localizer c-mods act to stop or rescue the spatial (re)positioning of a specific biomolecule without affecting most of a condensate’s integrity.

#### 2.3.1 Inhibition of aberrant transcriptional condensates in cancer

Localizer c-mods are therapeutic options for treating Acute Myeloid Leukaemia (AML). A frame shift in the nucleolar protein NPM1—a monogenic driver of AML, which accounts for ∼30% of cases globally—introduces an aberrant nuclear export signal (NES). This aberrant NES promotes strong interactions with Crm1/XPO1 (Xu et al., 2012), causing mutant NPM1 and a fraction of wild-type NPM1 to mislocalize from its cognate condensate, the nucleolus. The proteins ejected from the nucleolus reside in two distinct pools: 1) a cytoplasmic pool, generated by Crm1/XPO1-dependent nuclear export (Falini et al., 2006), and 2) a chromatin-bound pool, generated by direct recruitment *via* Crm1/XPO1 to *HOX* loci (Brunetti et al., 2018). Consequently, this condensatopathy is associated with a gain of function in the nucleus (reactivation of silenced *HOX* genes) (Brunetti et al., 2018) and loss of nucleolar function (nucleolar stress response) (Holoubek et al., 2021), leading to cellular transformation.

Small molecule localizer c-mods such as Avrainvillamide (Andresen et al., 2016) and Selinexor (KPT-330) (Table 1, shown and plotted in Figure 2A) (Gu et al., 2016; Brunetti et al., 2018; Holoubek et al., 2021), rescued NPM1 mislocalization, returning it to the nucleus and nucleolus, as well as restoring aspects of the gain and/or loss of function (reviewed in (Falini et al., 2006)). KPT-330 inhibits the interaction between mutant NPM1 and Crm1/XPO1 (Andresen et al., 2016; Gu et al., 2016; Brunetti et al., 2018). Despite the promise shown in preclinical and clinical studies (Ranganathan et al., 2012; Brunetti et al., 2018; Abboud et al., 2020; Sweet et al., 2021), this class of compounds inhibits nuclear export for all Crm1/XPO1 substrates, raising concerns of off-target effects. Development of localizer c-mods that can reverse the aberrant localization and misfunction of mutant NPM1 through mechanisms independent of the global function of Crm1/XPO1 could improve efficacy and reduce adverse effects, leading to superior drug candidates.

Localizer c-mods may also treat desmoplastic small round cell tumors (DSRCT), a rare and aggressive paediatric sarcoma with poor survival rates. In DSRCT, a genetic translocation leads to expression of an aberrant transcription factor EWS-WT1, which fuses the condensation-prone N-terminus of EWSR1 with the DNA-binding domain of WT1. Treatment with small molecule c-mod lurbinectedin (Table 1 and plotted in Figure 2A) leads to the localization of the fusion oncogene into the nucleolus, consequently supressing aberrant transcriptional activity, increasing cell viability, and improving survival rates in DSRCT mouse models (Gedminas et al., 2022). Lurbinectedin and its analog trabectedin (Table 1 and plotted in Figure 2A) are FDA-approved antineoplastic drugs used in the treatment of small cell lung cancer, and soft-tissue sarcoma and ovarian cancer, respectively. Interestingly, both compounds lead to nucleolar sequestration of EWS-FLI1 (a driver fusion oncogene in Ewing’s Sarcoma), inhibition of its transcriptional activity in cancer cells, and improved survival rate of xenograft mice (Harlow et al., 2016). The proposed mechanism of action is EWSR1 sequesteration in the nucleolus activates a DNA damage response. The lurbinectedin example illustrates a mechanism of action where a c-mod drives sequestration of a target protein inside a condensate to supress its aberrant function. Since both EWS-FLI1 and EWS-WT1 fusion oncogenes contain the N-terminus of EWSR1, this approach targets a central node shared between different clinical manifestations of sarcoma: aberrant transcription driven by the condensation of the N-terminus of EWSR1.

#### 2.3.2 Condensate-mediated drug resistance in cancer

Resistance to cancer therapies like cirzotinib against fusion oncogenic proteins, such as EML4-ALK in non-small cell lung cancer (NSCLC), can be explained by a localizer c-mod mechanism of action (Gonzalez-Martinez et al., 2022). EML4-ALK is a chimeric receptor tyrosine kinase, hyperactivation of which drives cancer. Multiple FDA-approved therapies exist for inhibiting the enzymatic activity of EML4-ALK. However, the existing therapies are efficacious initially, but suffer from disease relapse and drug resistance. Drug resistance to EML4-ALK-targeted therapies results in a post-therapy rescue of EGFR signaling. Activators of EGFR signaling are sequestered and inactivated by EML4-ALK condensates (Gonzalez-Martinez et al., 2022). Upon treatment with EML4-ALK inhibitor and localizer c-mod cirzotinib (Table 1 and shown in Figure 2A), the EGFR activators are re-localized away from the EML4-ALK condensate community resulting in an active EGFR signaling driven drug resistance. A clinical trial for the combined inhibition for EML4-ALK and EGFR by cirzotininb and erlotinib, respectively failed (Ou et al., 2017). Current understanding of the condensate-based mechanisms of EGFR signaling might allow us to discover additional specific inhibitors to target activators of the signaling pathway downstream of the current target of erlotinib, the kinase active site of EGFR (Gonzalez-Martinez et al., 2022). For example, a novel localizer c-mod may restrict the migration of EGFR activators from the EML4-ALK condensate.

#### 2.3.3 Condensates underlying specificity and toxicity of approved anti-cancer drugs

The SAR of localizer c-mods might explain the specificity and toxicity of some approved anti-cancer drugs. The nucleolus is the site of ribosome biogenesis and integrates cellular stress signaling. Chemotoxic stress induced by anti-cancer drugs actinomycin D (*via* inhibition of RNA synthesis) or MG132 (*via* proteasome activity) leads to changes in the function and biophysical properties of nucleoli. These changes have been observed as altered optical phenotype, mechanical properties (Louvet et al., 2014) and composition (Andersen et al., 2005), which ultimately result in arrested ribosome production and the initiation of several stress signaling pathways, including activation of p53-mediated apoptosis (reviewed in (Latonen, 2019)). A hallmark of nucleolar stress is the nucleoplasmic redistribution of NPM1, a component of the outer layer of the nucleolus. The closely related platinum compounds oxaliplatin, cisplatin and carboplatin are FDA-approved anti-neoplastic drugs and act as localizer c-mods; however, the latter two differ from oxaliplatin in the molecular mechanisms by which they induce cytotoxicity. Unlike the other two analogues which induce cellular toxicity *via* the DNA damage response, oxaliplatin acts as a c-mod by triggering a nucleolar stress response. Further SAR of 14 related platinum compound analogues, relative to their ability to affect NPM1 nucleolar/nucleoplasmic localization, uncovered a correlation between the c-mod potency and specific features of the compounds, such as steric bulk and hydrophobicity (Sutton et al., 2019). While the direct target(s) of these compounds are not fully elucidated, this study illustrates one of the first examples of a condensate-centric SAR; such approaches can be applied to design more efficacious and safer drugs in the future.

### 2.4 Morphers

Altering the microenvironment within a condensate and/or its material properties affects the activity of the condensate community (Alberti and Dormann, 2019). Changes in material properties can often be visualized in high content imaging screens as changes in morphology, including changes in size, distribution, or shape within the condensate. Morpher c-mods target condensate disease pathologies by altering the morphologies and material properties of condensates, which in turn affect their functions.

#### 2.4.1 Condensates supporting viral infection

Cyclopamine (Table 1 and plotted in Figure 2A) is a morpher c-mod that stops viral replication by solidifying a liquid-like replication condensate (Risso-Ballester et al., 2021). Replication of several infectious viruses are supported by underlying condensate-based mechanisms of the viroplasm in the infected host cell (Etibor et al., 2021). Replication of human respiratory syncytial virus (RSV) is enabled by a multi-phasic viroplasm. The outer phase concentrates replication-supporting factors and viral genomic RNA, while the inner phase is enriched in transcription factors and newly synthesized viral mRNA. Cyclopamine is a potent inhibitor of RSV replication with evidence from *in vivo* models (Risso-Ballester et al., 2021). However, the underlying mechanism of inhibition of RSV was attributed to the inhibition of the hedgehog pathway. A recent study showed that treatment with cyclopamine and analogues dissipate the transcription factor enriched sub-phase and solidify the surrounding viroplasm of the condensates. A single mutation in the transcription factor renders the condensates resistant to solidification by cyclopamine, which is confirmed by studies in cells and in mice (Risso-Ballester et al., 2021). Therefore, a transcription factor is the target of cyclopamine-driven solidification. The liquid properties of RSV condensates and dynamics of protein components are altered by cyclopamine within minutes, which further translates to efficacy in mice at relatively short time scales. Therefore, cylopamine acts as a morpher c-mod to alter the material properties of the RSV condensate, thereby inactivating a transcription factor and inhibiting viral replication.

Morpher c-mods offer a novel mechanism of action for drug discovery by harnessing the dynamic biophysical properties of condensates to affect the pathological mechanisms triggered by their components.

## 3 c-mod design principles

Notably, the chemical diversity of c-mods spans a wide breadth of chemical space and modalities (Table 1 and Figure 2). This supports the notion that specificity and selectivity can be rationally engineered to modulate condensate function. Selectivity and specificity can be achieved based on the following considerations.

Condensates differ from one another in their emergent properties, including composition and physical behavior. First, some condensates are enriched in RNA, while others are not. Second, the molecular grammar of the protein components (Wang et al., 2018; Kilgore and Young, 2022) provides a local environment with specific properties, such as pH, hydrophobicity, and enrichment in select binding sites and motifs. For example, the IDRs in some condensates are composed largely of aromatic residues, while in others they are highly charged or enriched in glycine or arginine residues. The level of ions and diverse cellular metabolites can also vary widely. Consequently, the physical environments within each condensate may be quite different, leading to preferential partitioning of compounds with specific chemical properties.

When a c-mod engages its target inside a condensate, optimizing the partitioning of the c-mod can have two synergistic advantages. First, it may increase the efficacy by promoting interactions with the intended target *via* increased local concentration of both target and ligand. Second, it minimizes off-target effects, as less of the c-mod is accessible outside the condensate. For example, the commonly used intercalating agent and chemotherapeutic cisplatin partitions more readily into transcriptional condensates *versus* other nuclear condensates. Thus, cisplatin preferentially binds and disrupts DNA associated with super enhancer-driven oncogenes compared to other sequences. This effect may direct cisplatin activity to relevant disease-causing genes, limiting exposure to other areas of the genome (Kilgore and Young, 2022).

*Should single or multiple components within a condensate community be targeted?*

C-mods may have more than one mechanism of action for a target condensate, if they interact with one or more components of a condensate community, in diverse ways. For example, an ASO may interact with RNA, a peptide may disrupt an interaction between two condensate components, a small molecule may block a protein-protein or protein-RNA interaction, a molecular glue may increase the proximity of two proteins, or a small molecule may inhibit the enzymatic function of a protein.

*Do c-mods need to interact directly with the target(s) in condensates?*

*Will targeting pathways that regulate the condensation process be a suitable strategy?*

Modulation of condensate properties and function can be achieved in several ways, including, but not limited to the classes we introduced (dissolvers, inducers, localizers and morphers). C-mods can act directly with condensate components (direct c-mods) or upstream, on targets that modify condensate components, such as PTM enzymes (indirect c-mods) (Kilgore and Young, 2022; Mitrea et al., 2022).

Direct c-mods may change the partitioning of a biomolecule into a particular condensate. This effect can be achieved by preferential interaction of the c-mod with the target inside or outside the condensate (reviewed in ref. (Ruff et al., 2021)), leading to a shift in the concentration threshold for condensation. Alternatively, the c-mod interaction could preferentially stabilize a conformational state, leading to an increase or decrease in the condensation threshold. For example, the dissolver NIP-V peptide stabilizes a monomeric conformation of SARS2-NP, thereby destabilizing the condensate (Wang et al., 2021) (Figure 2B). At the other end of the spectrum, the inducer BI-3802 binds to the BTB domain of BCL6 and drives formation of higher-order assemblies by stabilizing a dimerization interface (Słabicki et al., 2020) (Figure 2D). In both cases, the c-mods alter the valency of the self-assembling biomolecules, with decreased and increased valency leading to condensate dissolution and induction, respectively. Because of the nature of condensates—they are complex, highly dynamic, and have diverse functions and compositions—a desired effect (dissolving, inducing, localizing, morphing) may be achieved *via* a bespoke molecular engagement strategy.

Examples of indirect c-mods are molecules that change the activity of regulatory factors, such as PTM enzymes, chaperones, or proteases, to name a few. Indirect c-mods may act without entering the condensate at all; for example, the c-mod could block an upstream event such as a PTM of a protein, thereby preventing the protein from forming a condensate or entering an existing condensate. These regulatory factors could modulate the conformational space of one or more molecular community members, fine-tuning or dramatically changing the critical concentration for condensation. A recent proteomic study showed that phosphorylation is a major regulator of protein association with condensates (Sridharan et al., 2022). Notably, one can intervene with indirect c-mods at multiple steps within a signaling cascade. For example, small molecule inhibitors of mTORC1 and mTOR, such as everolimus and AZD8055 (Table 1 and shown in Figure 2A), respectively, prevented formation of G3BP1-containing SGs under starvation and oxidative stress conditions (Heberle et al., 2019). Similar dissolver c-mod effects on the assembly of SGs were observed with wortmannin (Table 1), an inhibitor of PI3K, which is located upstream of mTOR (Heberle et al., 2019). Indirect c-mods offer a plethora of opportunities to intervene in condensate homeostasis, using well-established methods of drug discovery. Caution should be exercised to minimize off-target effects. We believe intervention points closer to the condensate would be preferred.

## 4 Discussion and future perspectives

We summarized the emerging evidence that many known drugs discovered by classical means exert their effects, at least in part, *via* biomolecular condensates. Such examples are of scientific and historical interest, but also yield a compelling thesis—that a deeper understanding of condensate biology will enable the intentional development of novel drugs with similar condensate-specific effects. The discovery of condensates has deepened our understanding of many known but poorly explained phenomena in biology by using well-established concepts from physics, and chemistry (Hyman and Simons, 2012). In recent years, this understanding has extended to diverse diseases, where condensates have explained previously vague cellular pathophysiology. We propose that a better mechanistic understanding of c-mod action and the thoughtful application of condensate science may enable improvements in existing drugs and the discovery of novel classes of drugs. Additionally, this new vantage point enables access to a new pool of targets, historically considered “undruggable,” including proteins with high levels of structural dynamics and RNA (reviewed in (Conti and Oppikofer, 2022; Mitrea et al., 2022)).

*What are the phenotypic readouts of condensates that can be used for drug screening?*

Although the examples discussed in this manuscript classified the c-mods based on optical phenotypes obtained from conventional fluorescence microscopy, condensate-targeted drug screening can incorporate multiple types of established and emerging methodologies, described in detail in (Mitrea et al., 2022). Regardless of the chosen phenotypic readout, it is important to establish a relevant and robust functional correlation (see Box 1). Considering the wide range of approaches that one may take in discovering c-mods, success will require an open mind and a broad perspective of the chemical space to be explored. While the examples in Figures 2A–D demonstrate that classical structure-based drug discovery strategies can be applied successfully to develop c-mods, new opportunities and rules will emerge (e.g., engaging IDRs, leveraging emergent properties, *etc.*), enabling drug hunters to tackle “undruggable” targets.

### 4.1 c-mod discovery and data analysis considerations

We anticipate that machine learning (ML) will play a significant role in c-mod discovery, in multiple respects. First, when running a cellular phenotypic high-throughput screen (HTS) one should anticipate discovering compounds that operate through multiple mechanisms. SAR studies will consequently be quite complex and comprised of multiple independent subsets of hits. Complex learning algorithms may be employed to identify these independent chemical series. Second, although annotated compounds with known mechanisms of action may provide clues, such tool compounds are rarely selective, especially when tested at higher concentrations typically used in HTS. For this reason, ML can help identify the true mechanism(s) of action of c-mods. For example, screening hits may bind to multiple targets which inhabit different condensates, leading to complex cellular phenotypes. ML will enable deconvolution of the causes of such complex behaviour by considering the observed condensate phenotypes, the composition of each condensate, and the likely promiscuity of the screening hits. Third, even in cases where the c-mod mechanism has been established, one should anticipate that the SAR will be more complex than usual either because multiple targets are involved in the mechanism of the c-mod (polypharmacology), or because the partitioning of the c-mod into a condensate adds an element of complexity beyond the question of cell permeability. As mentioned above, we anticipate that the environment of each condensate may be unique, and consequently generalizable rules of partitioning are not anticipated.

### 4.2 Structure-activity relationship considerations

We anticipate that in some c-mod discovery programs, a structure-guided approach may be of use. In some cases, this will not apply because the mechanism of action of the c-mod will remain unknown or because some condensate components (e.g., IDRs) do not adopt a stable structure. However, in cases where structure-based approaches may be employed, we anticipate they will speed up the discovery process. Naturally, care must be exercised because the high complexity of the intermolecular interactions within condensates may make interpretation of structural information more complex. For these reasons, even in such structure-assisted projects, the primary screening tool may remain cellular due to the complexity of the c-mod mechanism of action because a biochemical screen would not recapitulate the relevant interactions, dynamics, or environment that is operative within the condensate.

### 4.3 Therapy considerations: distinct advantages of c-mods

There are several advantages to treating disease with c-mods. First, c-mods can be effective combination therapies, especially for therapies that suffer from chemoresistance. Condensates can be an integrating node of several mechanisms leading to chemoresistance (see SI-2 and bortezomib example above (Liu et al., 2021)). Therefore, c-mods can be more potent than classical inhibitors, especially when combined with chimeric protein degradation strategies such as PROTACs and molecular glues (see the BI-3802 example above). Induction of condensates by concentrating the material targeted for proteostasis seems to be a physiological response for a cell to achieve effective compartmentalisation and to optimize the activity of ubiquitin ligases (Lei et al., 2021). Condensates enriched with an ubiquitinated molecular community are directed by specific molecular regulators for proteasomal or autophagic clearance (Lei et al., 2021). Screening specifically for inducer c-mods that promote ubiquitination of the condensate community could be an attractive screening strategy for protein degradation targets. Third, c-mods can be attractive candidates for acute treatment schemes since the condensate physiochemical properties can respond at faster timescales to perturbations (see the cyclopamine example for the acute treatment for RSV infection above).

Taken together, we envision condensate science and c-mods will have a significant impact on the next‐generation of therapeutic development for several reasons. Condensates provide the opportunity to design more relevant screening strategies to integrate multiple disease-causing mechanisms into a single measurable read-out. C-mods span several modalities from small molecules to oligonucleotides and peptides, enabling more possibilities for drug discovery. Finally, c-mods offer the possibility to drive SAR of complex mechanisms by leveraging the power of machine learning and advance future discoveries.
